# Letter to the Editor: Safety of combination therapy of azilsartan medoxomil and amlodipine: a population-based cohort study

**DOI:** 10.4178/epih.e2025053

**Published:** 2025-09-20

**Authors:** Zhanyi Zhou

**Affiliations:** Zhejiang Chinese Medical University, Hangzhou, China

Dear Editor,

We read with great interest the recent article by Lee et al. [1], “Safety of combination therapy of azilsartan medoxomil and amlodipine: a population-based cohort study.” The authors are to be commended for their rigorous real-world evaluation, which included adjustment for numerous comorbidities such as chronic liver disease, chronic obstructive pulmonary disease, diabetes, and cerebrovascular and coronary artery disease. Nonetheless, we note that heart failure was not included among the baseline comorbidities examined.

Although the absence of a heart failure subgroup analysis may not invalidate the overall conclusions, it remains clinically important. In patients with hypertension and concomitant heart failure, who frequently have compromised hemodynamics, angiotensin II receptor blocker (ARB) therapy alone has been shown in randomized trials to increase the risk of hypotension by approximately 63% compared with placebo [2]. This concern may be particularly relevant for azilsartan medoxomil, a novel ARB with greater blood pressure–lowering potency than many older agents. When considering whether its safety profile truly parallels that of other ARBs, the potential for more pronounced blood pressure reduction in patients with hypertension and concomitant heart failure should be taken into account, as such patients may be especially susceptible to symptomatic hypotension and related complications.

Importantly, a systematic review reported that in symptomatic heart failure patients, ARB therapy was associated with a significantly higher risk of treatment discontinuation due to adverse events compared with control (prevalence ratio, 1.19; 95% confidence interval, 1.09 to 1.30), with hypotension being among the most common causes [3]. Furthermore, studies in older heart failure patients have demonstrated that valsartan use was linked to higher rates of hypotension, renal dysfunction, and hyperkalemia [4]. These findings underscore the importance of assessing this high-risk subgroup when evaluating the safety of ARB-based regimens.

Given that ARBs are central to both hypertension and guideline-directed heart failure management, explicitly assessing safety outcomes in the subgroup of patients with hypertension and concomitant heart failure would provide valuable guidance for clinical practice. We believe such analyses should not only be incorporated into secondary evaluations of the present study, but also be routinely considered in future safety research on other ARB-based regimens, to ensure that findings are generalizable across both the general population of individuals with hypertension and this higher-risk subgroup.

We appreciate the authors’ valuable contribution and hope that our suggestion will help refine the evidence base supporting the safe and effective use of ARBs in diverse patient populations.
